# 75200 Fecal Microbiota Transplantation to Prevent Infections in Patients with Acute Myeloid Leukemia: A Double-Blind Randomized Placebo-Controlled Phase 2 Clinical Trial

**DOI:** 10.1017/cts.2021.497

**Published:** 2021-03-30

**Authors:** Armin Rashidiryam Ebadi, Tauseef Ur Rehman, Harika Nalluri, Thomas Kaiser, Shernan G. Holtan, Alexander Khoruts, Daniel J. Weisdorf, Christopher Staley

**Affiliations:** University of Minnesota

## Abstract

ABSTRACT IMPACT: By restoring the gut microbiota in patients with acute myeloid leukemia exposed to antibiotics, we will reduce infections during and after curative-intent chemotherapy. OBJECTIVES/GOALS: Infection is a leading cause of death in acute myeloid leukemia (AML). Antibiotics disrupt the gut microbiota, promoting secondary infections. Through a double-blind randomized placebo-controlled phase 2 trial, we will determine whether microbiota restoration using fecal microbiota transplantation (FMT) prevents infections in AML patients. METHODS/STUDY POPULATION: 72 intensively treated AML patients at our institution are randomized in a 2:1 ratio to FMT (arm A) or placebo (arm B). After completing each course of antibacterial antibiotics, patients receive one study treatment. Up to 3 study treatments are administered over 3 months. FMT is delivered as a third-party oral product containing microbiota (˜5x10^11 bacteria) in 4-6 capsules. Stool samples are collected before and after each study treatment. The primary endpoint is 4-month overall infection rate. 16S rRNA gene sequencing of stool samples is used to determine specific taxa that are under- or over-represented in samples preceding infections and compare the two arms for key microbiome features including diversity and composition. Bloodstream infection within 7 days after FMT counts towards stopping rule. RESULTS/ANTICIPATED RESULTS: Five patients have been enrolled: 4 have received 1 dose and 1 received 2 doses. The only adverse event (possibly related to study treatment) has been grade 1 abdominal pain in 1 patient. Notably, no bloodstream infection has occurred. All planned samples have been collected and are sequenced in batches. We expect arm A patients to experience fewer infections and fewer intestinal blooms of pathobionts, and both arms to experience intestinal blooms before specific infections. An interim efficacy analysis will be performed at half total enrollment. DISCUSSION/SIGNIFICANCE OF FINDINGS: Current supportive care during intensive chemotherapy is fundamentally anti-microbial and results in dysbiosis, with detrimental consequences. We will establish the evidence for FMT as a restorative strategy in AML patients. This is the first randomized placebo-controlled trial of repeated FMT, with potential implications to other cancers.

